# Memantine targets glutamate receptors in atrial cardiomyocytes to prevent and treat atrial fibrillation

**DOI:** 10.1038/s41421-022-00429-8

**Published:** 2022-08-02

**Authors:** Duanyang Xie, Ke Xiong, Xuling Su, Guanghua Wang, Luxin Wang, Qicheng Zou, Caihong Zhang, Yuting Cao, Yi Liu, Yi-Han Chen

**Affiliations:** 1 Department of Cardiology, Shanghai East Hospital, Tongji University School of Medicine, Shanghai, China; 2 Key Laboratory of Arrhythmias of the Ministry of Education of China, Tongji University School of Medicine, Shanghai, China; 3 Institute of Medical Genetics, Tongji University, Shanghai, China; 4 Jinzhou Medical University, Jinzhou, Liaoning China; 5 Department of Pathology and Pathophysiology, Tongji University School of Medicine, Shanghai, China; 6 Research Units of Origin and Regulation of Heart Rhythm, Chinese Academy of Medical Sciences, Shanghai, China

Dear editor,

Atrial fibrillation (AF), the most common sustained cardiac arrhythmia, can lead to severe consequences such as heart failure and stroke^1^. Recently, we have demonstrated that atrial cardiomyocytes express ionotropic glutamate receptors (iGluRs) in high abundance, inhibition of which can significantly reduce the incidence of AF and effectively block AF progression in an experimental AF model using isolated rat hearts^2^. However, to date, none of medicines targeting iGluRs has been prescribed in clinical AF treatment. Memantine is a commonly used drug in clinic for the treatment of Alzheimer’s disease by antagonizing iGluRs in neurons^3^. We therefore hypothesized that memantine may also exert an effect against AF by blocking iGluRs in atrial cardiomyocytes.

First, we assessed the effect of memantine on the prevention and termination of AF. To examine the preventive effect of memantine on AF, we constructed three in vitro rat AF models, covering three common clinical types of AF, i.e., stretch-induced AF, cholinergic AF and ischemia-induced AF^4^. As shown in Fig. 1a, memantine perfusion effectively reduced the incidence of AF in a concentration-dependent manner. Based on the concentration-effect curve, we chose 100 μM as the optimal concentration of memantine for the following experiments, and the AF incidence in vehicle group versus 100 μM memantine group is as follows: 100% versus 0% in the stretch-induced AF model, 100% versus 0% in the cholinergic AF model and 90.9% versus 0% in the ischemia-induced AF model. The AF duration in the memantine group was also markedly shortened compared with that in the vehicle group (vehicle group versus memantine group: 47.5 ± 12.6 s versus 0 s in the stretch-induced AF model, 86.8 ± 20.9 s versus 0 s in the cholinergic AF model, and 49.4 ± 15.3 s versus 0 s in the ischemia-induced AF model). Then, we established three in vivo AF rat models to investigate the termination effect of memantine on AF: (1) a transverse aortic constriction (TAC)-induced AF model; (2) a cholinergic AF model by an intravenous bolus injection of acetylcholine (1 mg/kg, 0.1 mL) within 5 s; (3) an asphyxia-induced AF model by rapid atrial pacing during brief episodes of asphyxia. Electrocardiogram (ECG) recordings showing episodes of AF lasting more than 2 min were observed in all rats from all in vivo AF models. Once AF was successfully induced, the rats were immediately administered a single intravenous bolus injection of memantine (2.5 mg/kg, 0.1 mL) within 5 s. Figure 1b showed that memantine rapidly terminated AF within 2 min (termination rates in vehicle group versus memantine group: 0% versus 83.3% in the TAC-induced AF model, 0% versus 88.9% in the cholinergic AF model, and 0% versus 87.5% in the asphyxia-induced AF model). The AF duration in the memantine group was also markedly shortened compared with that in the vehicle group (vehicle group versus memantine group: 157.8 ± 11.4 s versus 55.3 ± 17.4 s in the TAC-induced AF model, 226.5 ± 47.3 s versus 70.4 ± 9.8 s in the cholinergic AF model, and 173.6 ± 21.1 s versus 64.2 ± 11.7 s in the asphyxia-induced AF model). These data strongly suggest that memantine can effectively prevent and terminate AF in a variety of animal models.

**Fig. 1:**
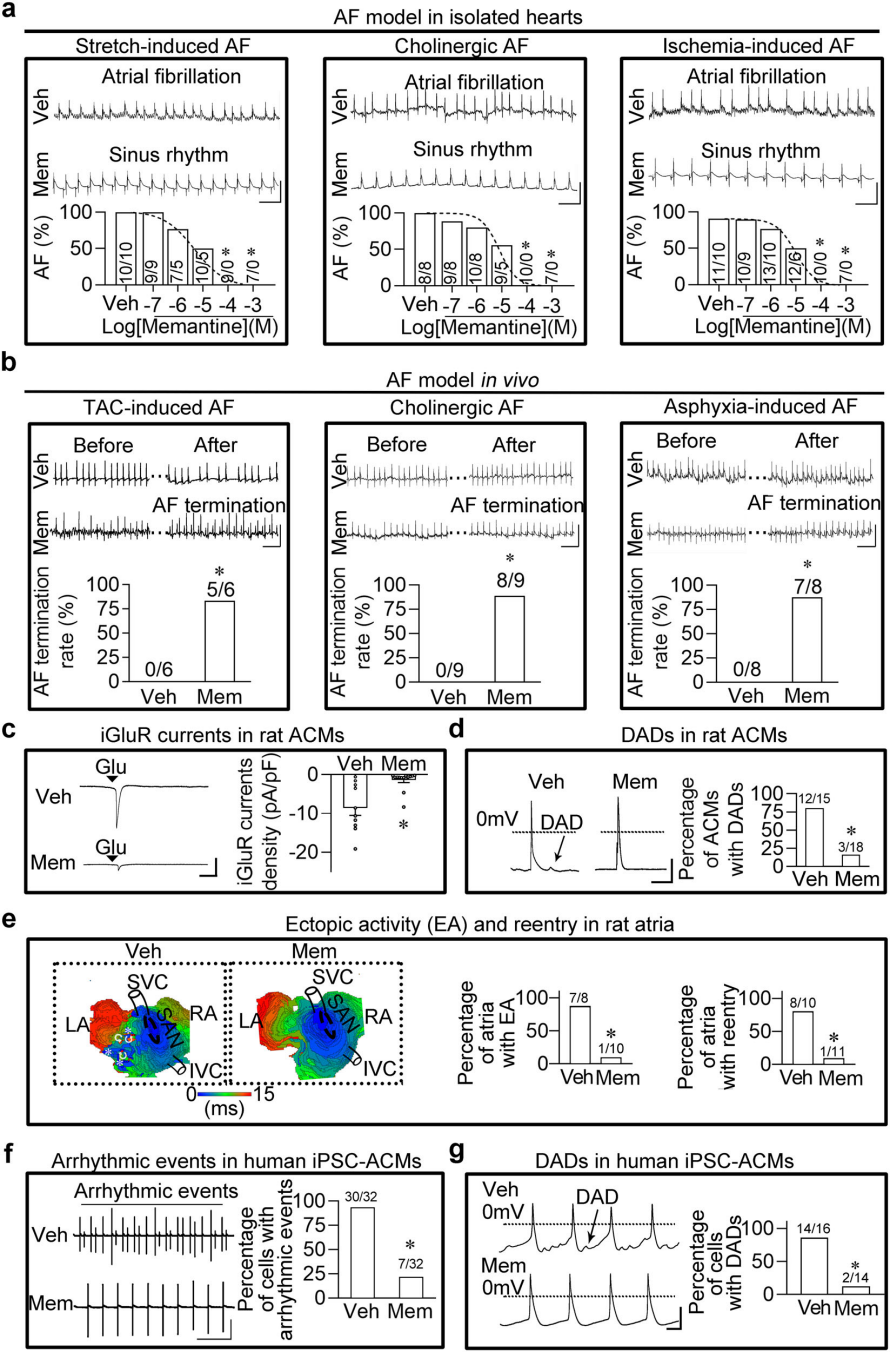
Memantine prevents and terminates AF in a variety of AF models by inhibiting iGluR currents in atrial cardiomyocytes. **a** Representative ECG traces of AF episodes (top), and quantification of the incidence of AF in the vehicle group and memantine group in three in vitro rat AF models (bottom; *P < 0.05, calculated by Chi-squared test, *n* = 7−13 rats at each concentration). Vertical scale bar, 5 mV; horizontal scale bar, 400 ms. **b** Representative ECG traces of AF episodes (top), and quantification of the termination rate of AF in the vehicle group and memantine group in three in vivo rat AF models (bottom; *P < 0.05, calculated by Chi-squared test, *n* = 6−9 rats per group per AF model). Vertical scale bar, 1 mV; horizontal scale bar, 500 ms. **c** Representative traces of an iGluR current in rat atrial cardiomyocytes induced by a 1 mM glutamate puff under voltage clamping at −60 mV (left). The bar graph showing iGluR current density in the vehicle group and memantine group (right; *P < 0.05, calculated by Student’s *t*-test, *n* = 10−14 cells per group). Vertical scale bar, 4 pA/pF; horizontal scale bar, 100 ms. **d** Typical traces of membrane potential in rat atrial cardiomyocytes treated with vehicle or 100 μM memantine (left). Pooled data of the percentage of ACMs with DADs from the left (right; *P < 0.05, calculated by Chi-squared test, *n* = 15−18 cells per group). Vertical scale bar, 30 mV; horizontal scale bar, 250 ms. **e** Representative optical mapping of the rat atria, and quantification of the incidence of ectopic activity and reentry in the vehicle group and memantine group (*P < 0.05, calculated by Chi-squared test, *n* = 8−11 rats per group). **f** Representative field potential traces of human iPSC-ACMs and quantification of the results (*P < 0.05, calculated by Chi-squared test, *n* = 32 per group). Vertical scale bar, 400 mV; horizontal scale bar, 1000 ms. **g** Representative membrane potential traces of DADs in single human iPSC-ACM and quantitative analysis of percentage of cells with DADs (*P < 0.05, calculated by Chi-squared test, *n* = 14−16 per group). Vertical scale bar, 50 mV; horizontal scale bar, 250 ms. Veh vehicle, Mem memantine, TAC transverse aortic constriction, Glu glutamate, ACMs atrial cardiomyocytes, SVC superior vena cava, IVC inferior vena caca, LA left atrium, RA right atrium, SAN sinoatrial node, DADs delayed afterdepolarizations.

Second, we analyzed how memantine affects the onset and progression of AF. To this end, we conducted four experiments. (1) In the central nervous system, memantine targets neuronal iGluRs^3^, but whether it targets iGluRs of atrial cardiomyocytes is still unknown. To answer this question, we first measured the effect of memantine on iGluR-gated currents (we call iGluR currents for short) in atrial cardiomyocytes. As shown in Fig. 1c, 100 μM memantine significantly inhibited the iGluR current (current density was inhibited from 8.53 ± 1.97 pA/pF to 1.4 ± 0.61 pA/pF). (2) It is well established that overactivation of iGluRs in neurons triggers excessive Ca^2+^ entry-induced Ca^2+^ overload^5^, and memantine can alleviate Ca^2+^ overload by suppressing the iGluRs^6^. Thus, our second experiment assessed the effect of memantine on Ca^2+^ leakage in atrial cardiomyocytes from the AF rats, showing 100 μM memantine significantly decreased the Ca^2+^ spark frequency (Supplementary Fig. S1). (3) Ca^2+^ leakage is associated with aberrant electrophysiological events that induce and maintain AF, such as abnormal spontaneous firing (ectopic activity) and atrial reentry^7,8^. To evaluate the role of memantine in these Ca^2+^ leakage-mediated events, we performed optical mapping which showed that 100 μM memantine reduced not only the incidence of ectopic activity (from 87.5 to 10%) in the isolated rat atrial myocardium subjected to 5 Hz pacing and hypoxia, but also the incidence of reentry (from 80% to 9.1%) in the isolated atrial myocardium from the AF rats (Fig. 1e). (4) Ca^2+^ leakage also contributes to delayed afterdepolarizations (DADs), one of the important sources of triggered activity or ectopic activity^9,10^. Based on this, we explored whether memantine affects DADs of atrial cardiomyocytes from the AF rats. Our patch-clamp data revealed that 100 μM memantine did decrease the percentage of atrial cardiomyocytes with DADs (from 80% to 16.6%) in rat atrial cardiomyocytes challenged with 2 Hz pacing stimulation (Fig. 1d). This series of experiments demonstrated that in atrial cardiomyocytes, memantine can also target iGluRs, thereby inhibiting iGluR currents-induced Ca^2+^ leakage, reducing or eliminating abnormal electrophysiological events, and ultimately leading to AF termination.

Finally, we evaluated the potential effect of memantine on aberrant electrophysiological events associated with AF in human with human induced pluripotent stem cell-derived atrial cardiomyocytes (iPSC-ACMs). By using a multielectrode array, we revealed that 50 μM memantine effectively decreased the percentage of cells with arrhythmic events from 93.8% to 21.9% (Fig. 1f). The patch-clamp data further showed that 50 μM memantine dramatically decreased the incidence of DADs (from 87.5% to 14.3%) in human iPSC-ACMs (Fig. 1g).

In summary, memantine can effectively prevent and terminate AF by blocking endogenous iGluRs in atrial cardiomyocytes, providing a new strategy for the clinical treatment of AF. Memantine has been widely prescribed for the treatment of Alzheimer’s disease^3^. For the first time, we here showed the beneficial effects of memantine on animal AF models. The new application of old medicines may greatly shorten the process of clinical drug development. We expect that memantine will eventually be clinically proven to be effective in the prevention and termination of AF, however, for the first step, clinical trials of memantine for AF are urgently needed.

## Supplementary information

Supplementary information
