# Supplementary material for: Memantine targets glutamate receptors in atrial cardiomyocytes to prevent and treat atrial fibrillation
Source: Cell Discov. 2022 Aug 2;8:76. doi: 10.1038/s41421-022-00429-8 (PMC9345967; doi:10.1038/s41421-022-00429-8)
Supplement: Supplementary file 1 — Supplementary information [file 41421_2022_429_MOESM1_ESM.pdf]

Supplementary materials

Materials and methods

Animals

This study was performed in accordance with the Guide for the Care and Use of Laboratory Animals of the U.S. National Institutes of Health. All animal experimental protocols were approved by the Institutional Animal Care and Use Committee of Tongji University School of Medicine.

***In vitro* atrial fibrillation (AF) induction**

Adult male Sprague–Dawley rats were anesthetized by intraperitoneal injection of pentobarbital (25 mg/kg) containing 120 IU heparin. The hearts were quickly excised and placed in oxygenated Tyrode's solution containing (in mM) 140 NaCl, 5.4 KCl, 1.8 $CaCl_2$, 1.2 KH_2PO_4, 5 HEPES, 1 $MgCl_2$, and 5.5 glucose (pH adjusted to 7.4 with NaOH). After washing out blood from the heart chambers, the hearts were connected to a Langendorff setup and perfused with 37°C oxygenated Tyrode's solution. For ECG recording of perfused hearts, electrocardiographic electrodes were attached to the free wall of the outside of the atria and screwed into the ventricular apex. The cardiac electrical signals were amplified and acquired with BioAmp and PowerLab (AD Instruments, USA). The data were analyzed manually using LabChart7 (AD Instruments, USA). All hearts were kept at 37 °C during the whole course of recording. AF was defined as the occurrence of rapid irregular atrial rhythms with irregular R-R intervals. The duration of AF was measured from the end of burst pacing to the first P-wave detected after the rapid irregular atrial rhythm.

A stretch-induced AF model was established as previously described with modifications[1]. Briefly, acute atrial dilation was conducted by ligating the inferior vena cava and then adjusting the perfusion speed to increase the atrial volume to twice the initial volume. After 40 min of atrial stretching, AF was induced by 20 Hz burst atrial pacing (pulse width 2 ms) for 40 seconds followed by 10 cycles of 40 seconds atrial pacing at 1-minute intervals.

A cholinergic AF (acetylcholine-induced AF) model was induced as previously described with modifications[1,2]. Acetylcholine (2 μM) was administered to generate a

rat model of acetylcholine-induced AF. Rat hearts were infused *in vitro* with acetylcholine for approximately 10 min, and then AF was induced by 20 Hz burst atrial pacing (pulse width 2 ms) for 40 seconds followed by 10 cycles of 40 seconds atrial pacing at 1-minute intervals.

An ischemia-induced AF model was established as previously described with modifications[1]. In brief, atrial ischemia was induced by perfusing hearts with one-fifth of normal perfusion pressure throughout. At the same time, right atrial pressure was maintained to prevent collapse during ischemia. After 30 min of ischemia, AF was induced by 20 Hz burst atrial pacing (pulse width 2 ms) for 40 seconds followed by 10 cycles of 40 seconds atrial pacing at 1-minute intervals.

***In vivo* AF induction**

An asphyxia-induced rat AF model was induced by rapid atrial pacing during brief episodes of asphyxia as previously described with minor modifications[3]. The rats were anesthetized using 1.5% isoflurane, artificially respirated, and kept at 37°C. A pacing catheter (1.4F, Millar Instruments, USA) was inserted via the right jugular vein into the right atria. The pacing threshold was determined by incrementally increasing the voltage until atrial capture occurred. Pacing was then performed at 2× pacing threshold. The rats were subjected to asphyxia, which was induced by clamping the tracheal tube with a pair of hemostatic forceps at the end of the inspiratory cycle, for 30 seconds. Ten seconds after the beginning of asphyxia stimulation, 20 Hz burst pacing was performed at 2× threshold voltage for 30 seconds to trigger AF. AF was defined as rapid and fragmented atrial electrograms with an irregular ventricular rhythm lasting for at least 2 min immediately following burst pacing.

A cholinergic rat AF model was established by injection of acetylcholine and rapid atrial pacing. Briefly, a pacing catheter (1.4F, Millar Instruments, USA) was inserted into the right atria of each anesthetized rat. Then, the rats received a single intravenous bolus injection of acetylcholine (1 mg/kg, 0.1 mL) within 5 seconds. Next, 20 Hz burst pacing was performed at 2× threshold voltage for 30 seconds after acetylcholine administration to trigger AF.

A transverse aortic constriction (TAC)-induced rat AF model was established as

previously described with modifications[4,5]. A pacing catheter (1.4F, Millar Instruments, USA) was inserted into the right atrium of the rat, and 20 Hz burst pacing was performed at 2× threshold voltage for 30 seconds to trigger AF.

Isolation of adult rat atrial cardiomyocytes

Atrial cardiomyocytes were isolated from the Langendorff-perfused hearts of adult male Sprague Dawley rats as previously described with minor modifications[6]. Briefly, the heart was removed, mounted on a Langendorff apparatus and perfused with Tyrode's solution to wash out the blood. Then, the heart was perfused with Ca^{2+}-free Tyrode's solution buffer containing 1 mg/mL collagenase type II (Worthington, USA) and 1 mg/mL BSA for digestion at 37°C for 15 min. When the heart became flaccid, the atria were dissected out from the heart, cut into small pieces, and resuspended in modified Kraftbruhe (KB) solution containing (in mM) 100 potassium glutamate, 10 potassium aspartate, 25 KCl, 10 KH_2PO_4, 2 $MgSO_4$, 20 taurine, 5 creatine, 0.5 EGTA, 20 glucose, and 5 HEPES and 1 mg/mL BSA (pH adjusted to 7.2 with KOH). Then, the cell-containing solution was filtered with a 100-μm filter. For electrophysiological experiments, gradient recalcification of the acutely isolated cells was performed by administering 1 M Ca^{2+} solution every 30 min until a final concentration of 1.8 mM was reached.

Patch-clamp recording

Whole-cell patch-clamp recording of atrial cardiomyocytes was performed as previously described[7]. Two borosilicate glass microelectrodes were used: one for electrical stimulation and recording with 2–5 MΩ resistances filled with the intracellular solution and the other for pulsatile administration with a tip of 10 μm inner diameter filled with the bath solution or bath solution containing glutamate (1 mM). Glutamate was delivered locally via the patch pipette connected to a PL1–100 picoinjector microinjector (Harvard Apparatus, USA). The iGluR current was recorded in voltage-clamp mode at a holding potential of −60 mV. APs were recorded in current-clamp mode, and all the data were collected with an EPC-10 amplifier (HEKA, Germany) according to a standard patch-clamp technique. The intracellular solution included (in mM): 140 KCl, 10 EGTA, 10 HEPES, 5 glucose and 3 Na_2ATP (pH

adjusted to 7.2 with KOH). Atrial cardiomyocytes were bathed in a chamber continuously perfused with oxygenated Tyrode's solution. All the data were analyzed with Patchmaster 2.42 (HEKA, Germany).

Ca^{2+} imaging

In brief, isolated atrial cardiomyocytes were loaded in 1 mL Tyrode's solution with 1 μM Cal-520 AM (AAT bioquest, USA) and 0.04% Pluronic F-127 (Sigma, USA) for 15 min. For Ca^{2+} confocal imaging, images were obtained with an Sp8 confocal microscope (Leica, USA) with a 63× oil objective (NA 1.4). The intensity of fluorescence was measured at Ex/Em = 488/515 nm. Spontaneous Ca^{2+} sparks and Ca^{2+} transients were recorded using line scan mode and were obtained at a rate of 1 ms per line. Data acquisition and analysis were performed using LAS X software (Leica, USA) and ImageJ 2.0 software (NIH, USA).

Optical mapping

Optical mapping experiments were performed essentially as described previously[8]. In brief, adult male Sprague–Dawley rats were anesthetized by intraperitoneal injection of pentobarbital (25 mg/kg) containing 120 IU heparin, the chest was opened, and the heart was harvested. After rapid excision and Langendorff perfusion at 37 °C with oxygenated Tyrode's solution, the heart was allowed to recover for 10 min and then loaded with the voltage-sensitive dye RH237 (10 μM, AAT bioquest, USA) for 5 min. Blebbistatin (10 μM, MCE, USA) was added to the perfusate to prevent motion artifacts. The dye was excited using LED light sources centered at 550 nm. Images were captured with a high-speed camera (MiCAM ULTIMA, USA). Activation time was determined as the time point of maximum change in fluorescence over time (dF/dt) for each fluorescent signal in the array. Reentry cycles were defined as repetitive returns of an impulse into the same atrial area with a constant rotation pattern over at least 2 cycles. All the data were collected and analyzed using MiCAM ULTIMA acquisition software and BV_Ana software (SciMedia, USA).

Multielectrode array (MEA) recording

Human induced pluripotent stem cell-derived atrial cardiomyocytes (iPSC-ACMs) were obtained from the Institute of Biophysics of the Chinese Academy of Sciences

(Beijing, China). After recovery, the cells were cultured in a T25 flask precoated with vitronectin (0.01 μg/μL, Cauliscell, China) in cardiomyocyte maintenance medium (Cauliscell, China) at 37°C and 5% CO_2. The medium was refreshed every 2 days.

At day 12, the cells were dissociated and resuspended in cardiac recovery medium at a density of 3 x 10^6 cells/mL. Aliquots of the cell suspensions (10 μL) were plated on CytoView MEA 24-well plates (Axion BioSystems, USA). The MEA device automatically adjusted and controlled the environment (37°C and 5% CO_2) to maintain the temperature and pH of the medium. The data were acquired using Maestro MEA System (Axion BioSystems, USA). The induction of arrhythmic events on MEA recording was carried out as previously reported with some modification[9,10], human iPSC-ACMs were treated with 50 μM sotalol and cultured in a nitrogen-supplied hypoxia incubator subjected to a cycle of hypoxia (1% oxygen) for 3 h and overnight normoxia (19% oxygen).

Western blotting

The protein was extracted by RIPA lysis buffer (Beyotime Biotechnology, China) supplemented with a cocktail of protease inhibitors and phosphatase inhibitors (Roche, USA) at 4°C. Protein concentrations were determined with BCA kit (Beyotime Biotechnology, China), and equal amount of protein was separated by 10% SDS-PAGE (Thermo Fisher Scientific, USA), then transferred onto PVDF membranes (Millipore, USA). Next, the membranes were blocked with 5% BSA in TBS + 0.1% Tween (TBST) for 1 h, then incubated with primary antibody against RyR2 (Abcam, USA), RyR2 (phospho-S2814) (Badrilla, UK) or GAPDH (Abcam, USA) overnight at 4℃. The next day, the membranes were washed with TBST and incubated with the HRP-conjugated secondary antibody (Invitrogen, USA) for 1 h, and the bands were visualized with ChemiDoc Touch Gel Imaging System (Bio-Rad, USA).

Statistics

Data normality was tested using the Kolmogorov–Smirnov test. For comparisons between two groups, two-tailed Student's *t*-test or the nonparametric Mann–Whitney test was used. Statistics on percentages were performed with a Chi-squared test. Statistical significance was defined as $p \leq 0.05$. Each animal sample and drug used in

the electrophysiological experiments was assigned a code, and the data were unmasked upon completion of the study. All experiments were repeated at least 3 times. All the data were presented as the mean ± SEM. All statistical analyses were performed with GraphPad Prism 8.

Supplementary references

1. Li, J. et al. Inhibition of mitochondrial translocator protein prevents atrial fibrillation. *Eur J Pharmacol* **632**, 60-64 (2010).
2. Chong, E. et al. Resveratrol, a red wine antioxidant, reduces atrial fibrillation susceptibility in the failing heart by PI3K/AKT/eNOS signaling pathway activation. *Heart Rhythm* **12**, 1046-1056 (2015).
3. Haugan, K., Lam, H.R., Knudsen, C.B. & Petersen, J.S. Atrial fibrillation in rats induced by rapid transesophageal atrial pacing during brief episodes of asphyxia: a new in vivo model. *J Cardiovasc Pharmacol* **44**, 125-135 (2004).
4. Terrenoire, C., Lauritzen, I., Lesage, F., Romey, G. & Lazdunski, M. A TREK-1-like potassium channel in atrial cells inhibited by beta-adrenergic stimulation and activated by volatile anesthetics. *Circ Res* **89**, 336-342 (2001).
5. Messaoudi, S. et al. Aldosterone-specific activation of cardiomyocyte mineralocorticoid receptor in vivo. *Hypertension* ***61***, 361-367 (2013).
6. Zhang, Y. et al. Stretch-induced sarcoplasmic reticulum calcium leak is causatively associated with atrial fibrillation in pressure-overloaded hearts. *Cardiovasc Res* ***117***, 1091-1102 (2021).
7. Voigt, N. et al. Cellular and molecular mechanisms of atrial arrhythmogenesis in patients with paroxysmal atrial fibrillation. *Circulation* **129**, 145-156 (2014).
8. Hong, T. et al. Cardiac BIN1 folds T-tubule membrane, controlling ion flux and limiting arrhythmia. *Nat. Med* **20**, 624–632 (2014).
9. Navarrete, E.G. et al. Screening drug-induced arrhythmia using human induced pluripotent stem cell-derived cardiomyocytes and low-impedance microelectrode arrays. *Circulation* **128**, S3-13 (2013).
10. Shah, D. et al. Modeling of LMNA-Related Dilated Cardiomyopathy Using Human

Induced Pluripotent Stem Cells. *Cells* **8**, 594 (2019).

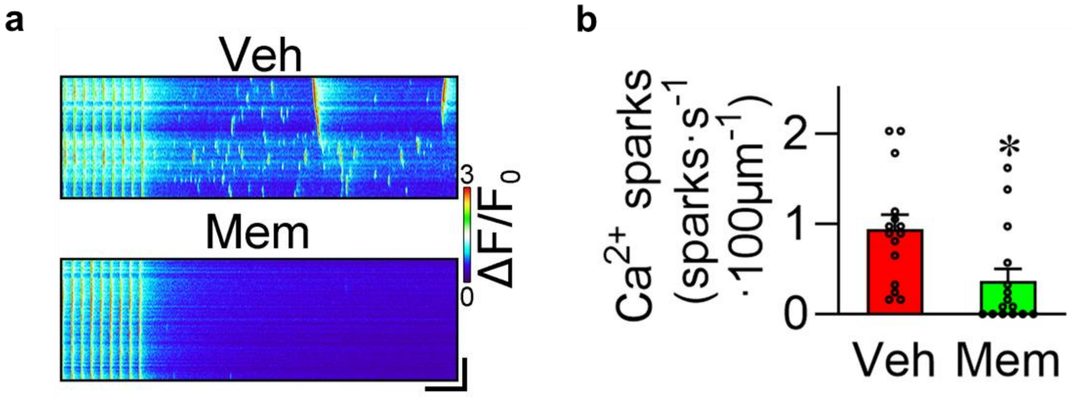

Supplementary Fig. S1. Memantine treatment attenuated Ca^{2+} leakage in atrial cardiomyocytes from the AF rats.

a Representative images of Ca^{2+} sparks in Cal-520-loaded atrial cardiomyocytes from rats with AF in the presence and absence of 100 μM Memantine. **b** Quantification of the Ca^{2+} spark frequency in the vehicle group and Memantine group (right; * $p < 0.05$, analyzed by the *t* test, n = 15 cells per group). Vertical scale bar, 20 μm; horizontal scale bar, 1000 ms.

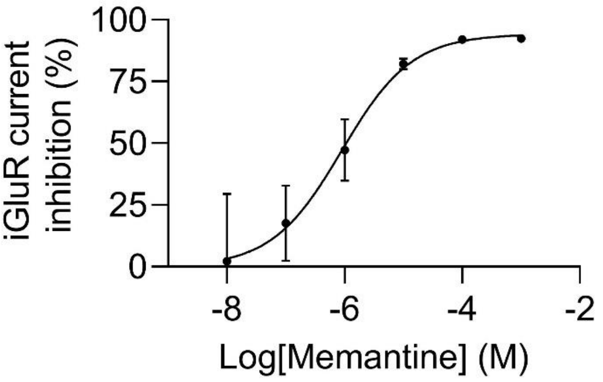

Supplementary Fig. S2. Memantine inhibited the iGluR current in a concentration-dependent manner.

Concentration-response curves demonstrating the dose-dependence of inhibitory effect of Memantine on the iGluR current in rat atrial cardiomyocytes (EC50 of Memantine = 0.89 μM). n = 6-8 at each concentration. The EC50 value was calculated using four parametric nonlinear regression.

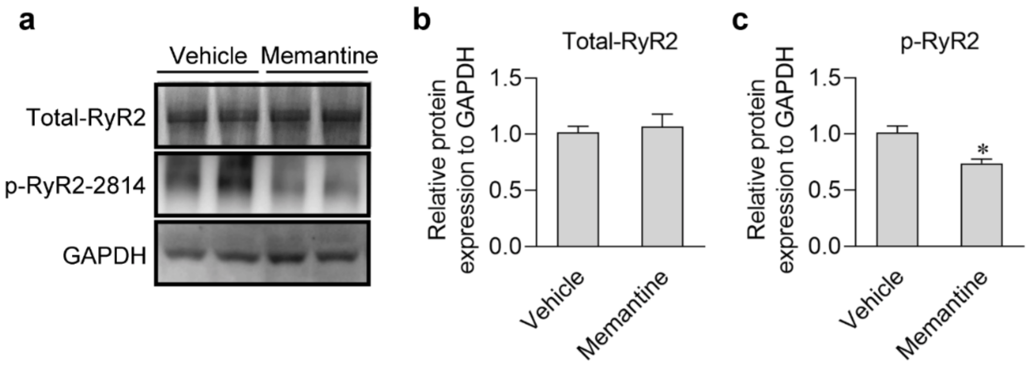

Supplementary Fig. S3. Memantine effectively decreased the phosphorylation level of RyR2 in the atria of the AF rats.

a. Representative western blot showing that application of 100 μM Memantine decreased phosphorylation of RyR2 (Ser-2814) but not the total RyR2 expression in the AF rats. **b-c.** Pooled data from **a**. * $p < 0.05$, analyzed by the *t* test, n = 6.
